# Machine learning using intrinsic genomic signatures for rapid classification of novel pathogens: COVID-19 case study

**DOI:** 10.1371/journal.pone.0232391

**Published:** 2020-04-24

**Authors:** Gurjit S. Randhawa, Maximillian P. M. Soltysiak, Hadi El Roz, Camila P. E. de Souza, Kathleen A. Hill, Lila Kari

**Affiliations:** 1 Department of Computer Science, The University of Western Ontario, London, ON, Canada; 2 Department of Biology, The University of Western Ontario, London, ON, Canada; 3 Department of Statistical and Actuarial Sciences, The University of Western Ontario, London, ON, Canada; 4 School of Computer Science, University of Waterloo, Waterloo, ON, Canada; Kliniken der Stadt Köln gGmbH, GERMANY

## Abstract

The 2019 novel coronavirus (renamed SARS-CoV-2, and generally referred to as the COVID-19 virus) has spread to 184 countries with over 1.5 million confirmed cases. Such major viral outbreaks demand early elucidation of taxonomic classification and origin of the virus genomic sequence, for strategic planning, containment, and treatment. This paper identifies an intrinsic COVID-19 virus genomic signature and uses it together with a machine learning-based alignment-free approach for an ultra-fast, scalable, and highly accurate classification of whole COVID-19 virus genomes. The proposed method combines supervised machine learning with digital signal processing (MLDSP) for genome analyses, augmented by a decision tree approach to the machine learning component, and a Spearman’s rank correlation coefficient analysis for result validation. These tools are used to analyze a large dataset of over 5000 unique viral genomic sequences, totalling 61.8 million bp, including the 29 COVID-19 virus sequences available on January 27, 2020. Our results support a hypothesis of a bat origin and classify the COVID-19 virus as *Sarbecovirus*, within *Betacoronavirus*. Our method achieves 100% accurate classification of the COVID-19 virus sequences, and discovers the most relevant relationships among over 5000 viral genomes within a few minutes, *ab initio*, using raw DNA sequence data alone, and without any specialized biological knowledge, training, gene or genome annotations. This suggests that, for novel viral and pathogen genome sequences, this alignment-free whole-genome machine-learning approach can provide a reliable real-time option for taxonomic classification.

## Introduction

Coronaviruses are single-stranded positive-sense RNA viruses that are known to contain some of the largest viral genomes, up to around 32 kbp in length [1–5]. After increases in the number of coronavirus genome sequences available following efforts to investigate the diversity in the wild, the family *Coronaviridae* now contains four genera (International Committee on Taxonomy of Viruses, [6]). While those species that belong to the genera *Alphacoronavirus* and *Betacoronavirus* can infect mammalian hosts, those in *Gammacoronavirus* and the recently defined *Deltacoronavirus* mainly infect avian species [4, 7–9]. Phylogenetic studies have revealed a complex evolutionary history, with coronaviruses thought to have ancient origins and recent crossover events that can lead to cross-species infection [8, 10–12]. Some of the largest sources of diversity for coronaviruses belong to the strains that infect bats and birds, providing a reservoir in wild animals for recombination and mutation that may enable cross-species transmission into other mammals and humans [4, 7, 8, 10, 13].

Like other RNA viruses, coronavirus genomes are known to have genomic plasticity, and this can be attributed to several major factors. RNA-dependent RNA polymerases (RdRp) have high mutation rates, reaching from 1 in 1000 to 1 in 10000 nucleotides during replication [7, 14, 15]. Coronaviruses are also known to use a template switching mechanism which can contribute to high rates of homologous RNA recombination between their viral genomes [9, 16–20]. Furthermore, the large size of coronavirus genomes is thought to be able to accommodate mutations to genes [7]. These factors help contribute to the plasticity and diversity of coronavirus genomes today.

The highly pathogenic human coronaviruses, Severe Acute Respiratory Syndrome coronavirus (SARS-CoV) and Middle East respiratory syndrome coronavirus (MERS-CoV) belong to lineage B (sub-genus *Sarbecovirus*) and lineage C (sub-genus *Merbecovirus*) of *Betacoronavirus*, respectively [9, 21–23]. Both result from zoonotic transmission to humans and lead to symptoms of viral pneumonia, including fever, breathing difficulties, and more [24, 25]. Recently, an unidentified pneumonia disease with similar symptoms caused an outbreak in Wuhan and is thought to have started from a local fresh seafood market [26–30]. This was later attributed to a novel coronavirus (the COVID-19 virus), and represents the third major zoonotic human coronavirus of this century [31]: On February 28, 2020, the World Health Organization set the COVID-19 risk assessment for regional and global levels to “Very High” [32].

From analyses employing whole genome to viral protein-based comparisons, the COVID-19 virus is thought to belong to lineage B (*Sarbecovirus*) of *Betacoronavirus*. From phylogenetic analysis of the RdRp protein, spike proteins, and full genomes of the COVID-19 virus and other coronaviruses, it was found that the COVID-19 virus is most closely related to two bat SARS-like coronaviruses, *bat-SL-CoVZXC21* and *bat-SL-CoVZC45*, found in Chinese horseshoe bats *Rhinolophus sinicus* [12, 33–37]. Along with the phylogenetic data, the genome organization of the COVID-19 virus was found to be typical of lineage B (*Sarbecovirus*) *Betacoronaviruses* [33]. From phylogenetic analysis of full genome alignment and similarity plots, it was found that the COVID-19 virus has the highest similarity to the bat coronavirus *RaTG13* [38]. Close associations to bat coronavirus *RaTG13* and two bat SARS-like CoVs (*ZC45* and *ZXC21*) are also supported in alignment-based phylogenetic analyses [38]. Within the COVID-19 virus sequences, over 99% sequence similarity and a lack of diversity within these strains suggest a common lineage and source, with support for recent emergence of the human strain [12, 31]. There is ongoing debate whether the COVID-19 virus arose following recombination with previously identified bat and unknown coronaviruses [39] or arose independently as a new lineage to infect humans [38]. In combination with the identification that the angiotensin converting enzyme 2 (ACE2) protein is a receptor for COVID-19 virus, as it is for SARS and other *Sarbecovirus* strains, the hypothesis that the COVID-19 virus originated from bats is deemed very likely [12, 33, 35, 38, 40–44].

All analyses performed thus far have been alignment-based and rely on the annotations of the viral genes. Though alignment-based methods have been successful in finding sequence similarities, their application can be challenging in many cases [45, 46]. It is realistically impossible to analyze thousands of complete genomes using alignment-based methods due to the heavy computation time. Moreover, the alignment demands the sequences to be continuously homologous which is not always the case. Alignment-free methods [47–51] have been proposed in the past as an alternative to address the limitations of the alignment-based methods. Comparative genomics beyond alignment-based approaches have benefited from the computational power of machine learning. Machine learning-based alignment-free methods have also been used successfully for a variety of problems including virus classification [49–51]. An alignment-free approach [49] was proposed for subtype classification of HIV-1 genomes and achieved ∼97% classification accuracy. MLDSP [50], with the use of a broad range of 1*D* numerical representations of DNA sequences, has also achieved very high levels of classification accuracy with viruses. Even rapidly evolving, plastic genomes of viruses such as *Influenza* and *Dengue* are classified down to the level of strain and subtype, respectively with 100% classification accuracy. MLDSP-GUI [51] provides an option to use 2*D* Chaos Game Representation (CGR) [52] as numerical representation of DNA sequences. CGR’s have a longstanding use in species classification with identification of biases in sequence composition [48, 51, 52]. MLDSP-GUI has shown 100% classification accuracy for *Flavivirus* genus to species classification using 2*D* CGR as numerical representation [51]. MLDSP and MLDSP-GUI have demonstrated the ability to identify the genomic signatures (a species-specific pattern known to be pervasive throughout the genome) with species level accuracy that can be used for sequence (dis)similarity analyses. In this study, we use MLDSP [50] and MLDSP-GUI [51] with CGR as a numerical representation of DNA sequences to assess the classification of the COVID-19 virus from the perspective of machine learning-based alignment-free whole genome comparison of genomic signatures. Using MLDSP and MLDSP-GUI, we confirm that the COVID-19 virus belongs to the *Betacoronavirus*, while its genomic similarity to the sub-genus *Sarbecovirus* supports a possible bat origin.

This paper demonstrates how machine learning using intrinsic genomic signatures can provide rapid alignment-free taxonomic classification of novel pathogens. Our method delivers accurate classifications of the COVID-19 virus without *a priori* biological knowledge, by a simultaneous processing of the geometric space of all relevant viral genomes. The main contributions are:

- Identifying intrinsic viral genomic signatures, and utilizing them for a real-time and highly accurate machine learning-based classification of novel pathogen sequences, such as the COVID-19 virus;
- A general-purpose bare-bones approach, which uses raw DNA sequences alone and does not have any requirements for gene or genome annotation;
- The use of a “decision tree” approach to supervised machine learning (paralleling taxonomic ranks), for successive refinements of taxonomic classification.
- A comprehensive and “in minutes” analysis of a dataset of 5538 unique viral genomic sequences, for a total of 61.8 million bp analyzed, with high classification accuracy scores at all levels, from the highest to the lowest taxonomic rank;
- The use of Spearman’s rank correlation analysis to confirm our results and the relatedness of the COVID-19 virus sequences to the known genera of the family *Coronaviridae* and the known sub-genera of the genus *Betacoronavirus*.

## Materials and methods

The Wuhan seafood market pneumonia virus (COVID-19 virus/SARS-CoV-2) isolate Wuhan-Hu-1 complete reference genome of 29903 bp was downloaded from the National Center for Biotechnology Information (NCBI) database on January 23, 2020. All of the available 28 sequences of COVID-19 virus and the bat *Betacoronavirus* *RaTG13* from the GISAID platform, and two additional sequences (*bat-SL-CoVZC45*, and *bat-SL-CoVZXC21*) from the NCBI, were downloaded on January 27, 2019. All of the available viral sequences were downloaded from the Virus-Host DB (14688 sequences available on January 14, 2020). Virus-Host DB covers the sequences from the NCBI RefSeq (release 96, September 9, 2019) and GenBank (release 233.0, August 15, 2019). All sequences shorter than 2000 bp and longer than 50000 bp were ignored to address possible issues arising from sequence length bias. Accession numbers for all the sequences used in this study can be found in S1 File of S2 and S3 Tables.

MLDSP [50] and MLDSP-GUI [51] were used as the machine learning-based alignment-free methods for complete genome analyses. As MLDSP-GUI is an extension of the MLDSP methodology, we will refer to the method hereafter as MLDSP-GUI. Each genomic sequence is mapped into its respective genomic signal (a discrete numeric sequence) using a numerical representation. For this study, we use a two-dimensional *k*-mer (oligomers of length *k*) based numerical representation known as Chaos Game Representation (CGR) [52]. The *k*-mer value 7 is used for all the experiments. The value *k* = 7 achieved the highest accuracy scores for the HIV-1 subtype classification [49] and this value could be relevant for other virus related analyses. The magnitude spectra are then calculated by applying Discrete Fourier Transform (DFT) to the genomic signals [50]. A pairwise distance matrix is then computed using the Pearson Correlation Coefficient (PCC) [53] as a distance measure between magnitude spectra. The distance matrix is used to generate the 3D Molecular Distance Maps (MoDMap3D) [54] by applying the classical Multi-Dimensional Scaling (MDS) [55]. MoDMap3D represents an estimation of the relationship among sequences based on the genomic distances between the sequences. The feature vectors are constructed from the columns of the distance matrix and are used as an input to train six supervised-learning based classification models (Linear Discriminant, Linear SVM, Quadratic SVM, Fine KNN, Subspace Discriminant, and Subspace KNN) [50]. A 10-fold cross-validation is used to train, and test the classification models and the average of 10 runs is reported as the classification accuracy. The trained machine learning models are then used to test the COVID-19 virus sequences. The unweighted pair group method with arithmetic mean (UPGMA) [56] and neighbor-joining [57] phylogenetic trees are also computed using the pairwise distance matrix.

In this paper, MLDSP-GUI is augmented by a decision tree approach to the supervised machine learning component and a Spearman’s rank correlation coefficient analysis for result validation. The decision tree parallels the taxonomic classification levels, and is necessary so as to minimize the number of calls to the supervised classifier module, as well as to maintain a reasonable number of clusters during each supervised training session. For validation of MLDSP-GUI results using CGR as a numerical representation, we use Spearman’s rank correlation coefficient [58–61], as follows. The frequency of each *k*-mer is calculated in each genome. Due to differences in genome length between species, proportional frequencies are computed by dividing each *k*-mer frequency by the length of the respective sequence. To determine whether there is a correlation between *k*-mer frequencies in COVID-19 virus genomes and specific taxonomic groups, a Spearman’s rank correlation coefficient test is conducted for *k* = 1 to *k* = 7.

## Results

Table 1 provides the details of three datasets Test-1, Test-2, Test-3a and Test-3b used for analyses with MLDSP-GUI. Each dataset’s composition (clusters with number of sequences), the respective sequence length statistics, and results of MLDSP-GUI after applying 10-fold cross-validation as classification accuracy scores are shown. The classification accuracy scores for all six classification models are shown with their average, see Table 1.

**Table 1:** Classification accuracy scores of viral sequences at different levels of taxonomy.

Dataset	Clusters	Number of sequences	Classification model	Classification accuracy (in %)
Test-1: 11 families and Riboviria; 3273 sequences; Maximum length: 49973 Minimum length: 2002 Median length: 7350 Mean length: 13173	*Adenoviridae* *Anelloviridae* *Caudovirales* *Geminiviridae* *Genomoviridae* *Microviridae* *Ortervirales* *Papillomaviridae* *Parvoviridae* *Polydnaviridae* *Polyomaviridae* Riboviria	198 126 500 500 115 102 233 369 182 304 144 500	LinearDiscriminant LinearSVM QuadraticSVM FineKNN SubspaceDiscriminant SubspaceKNN AverageAccuracy	91.7 90.8 95 93.4 87.6 93.2 92
Test-2: Riboviria families; 2779 sequences; Maximum length: 31769 Minimum length: 2005 Median length: 7488 Mean length: 8607	Betaflexiviridae Bromoviridae Caliciviridae Coronaviridae Flaviviridae Peribunyaviridae Phenuiviridae Picornaviridae Potyviridae Reoviridae Rhabdoviridae Secoviridae	121 122 403 210 222 166 107 437 196 470 192 133	LinearDiscriminant LinearSVM QuadraticSVM FineKNN SubspaceDiscriminant SubspaceKNN AverageAccuracy	91.2 89.2 93.1 90.3 89 90.4 90.5
Test-3a: *Coronaviridae*; 208 sequences; Maximum length: 31769 Minimum length: 9580 Median length: 29704 Mean length: 29256	Alphacoronavirus Betacoronavirus Deltacoronavirus Gammacoronavirus	53 126 20 9	LinearDiscriminant LinearSVM QuadraticSVM FineKNN SubspaceDiscriminant SubspaceKNN AverageAccuracy	98.1 94.2 95.2 95.7 97.6 96.2 96.2
Test-3b: *Coronaviridae*; 60 sequences; Maximum length: 31429 Minimum length: 25402 Median length: 28475 Mean length: 28187	Alphacoronavirus Betacoronavirus Deltacoronavirus	20 20 20	LinearDiscriminant LinearSVM QuadraticSVM FineKNN SubspaceDiscriminant SubspaceKNN AverageAccuracy	100 93.3 93.3 95 95 95 95.3

All classifiers trained on Test-1, Test-2, Test-3a, and Test-3b datasets were used to predict the labels of 29 COVID-19 virus sequences. All classifiers predicted the correct labels for all of the sequences (Riboviria when trained using Test-1, *Coronaviridae* when trained using Test-2, and *Betacoronavirus* when trained using Test-3a and Test-3b).

**Fig 1:**
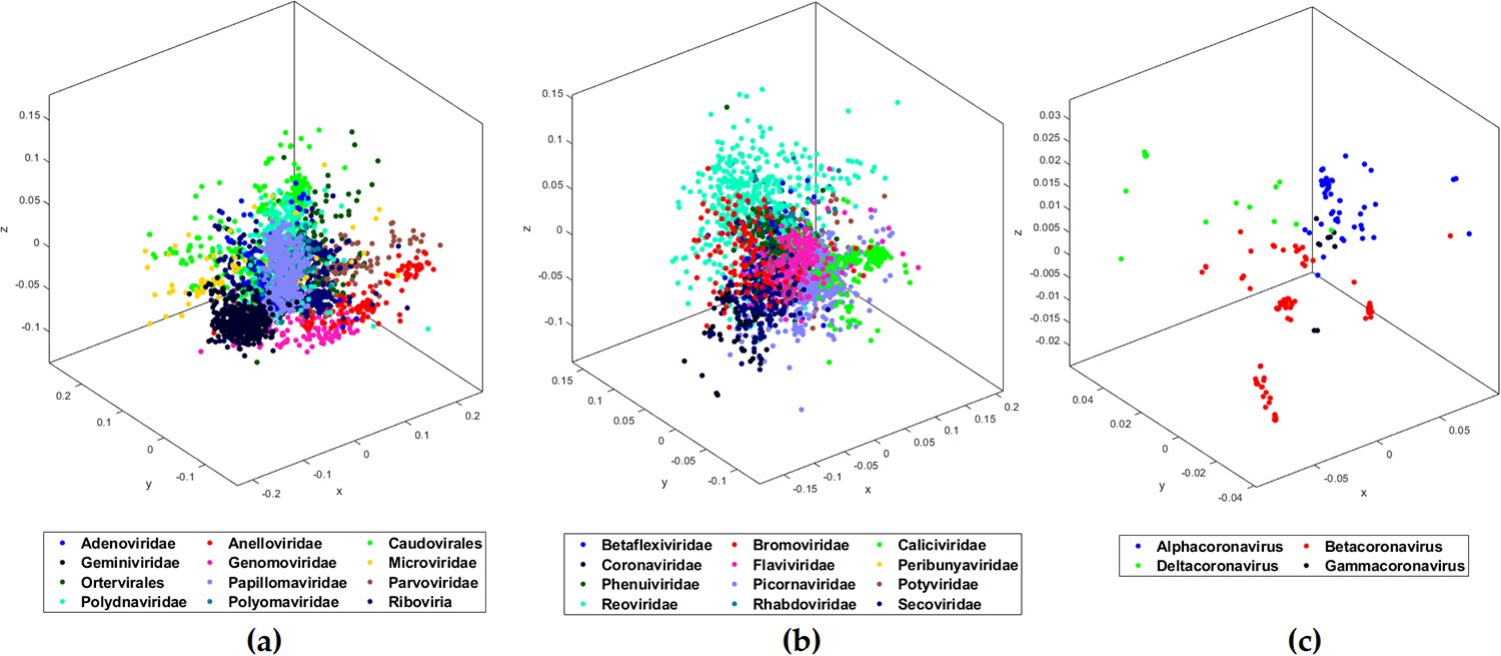
MoDMap3D of (a) 3273 viral sequences from Test-1 representing 11 viral families and realm Riboviria, (b) 2779 viral sequences from Test-2 classifying 12 viral families of realm Riboviria, (c) 208 *Coronaviridae* sequences from Test-3a classified into genera.

As shown in Table 1, for the first test (Test-1), we organized the dataset of sequences into 12 clusters (11 families, and Riboviria realm). Only the families with at least 100 sequences were considered. The Riboviria cluster contains all families that belong to the realm Riboviria. For the clusters with more than 500 sequences, we selected 500 sequences at random. Our method can handle all of the available 14668 sequences, but using imbalanced clusters, in regard to the number of sequences, can introduce an unwanted bias. After filtering out the sequences, our pre-processed dataset is left with 3273 sequences organized into 12 clusters (*Adenoviridae*, *Anelloviridae*, *Caudovirales*, *Geminiviridae*, *Genomoviridae*, *Microviridae*, *Ortervirales*, *Papillomaviridae*, *Parvoviridae*, *Polydnaviridae*, *Polyomaviridae*, and Riboviria). We used MLDSP-GUI with CGR as the numerical representation at *k* = 7. The maximum classification accuracy of 94.9% is obtained using the Quadratic SVM model. The respective MoDMap3D is shown in Fig 1(a). All six classification models trained on 3273 sequences were used to classify (predict the labels of) the 29 COVID-19 virus sequences. All of our machine learning-based models correctly predicted and confirmed the label as Riboviria for all 29 sequences (Table 2).

**Table 2:** Predicted taxonomic labels of 29 COVID-19 virus sequences.

Training dataset	Testing dataset	Classification models	Prediction accuracy (%)	Predicted label
Test-1	29 COVID-19 virus sequences	Linear Discriminant Linear SVM Quadratic SVM Fine KNN Subspace Discriminant Subspace KNN	100 100 100 100 100 100	Riboviria Riboviria Riboviria Riboviria Riboviria Riboviria
Test-2	29 COVID-19 virus sequences	Linear Discriminant Linear SVM Quadratic SVM Fine KNN Subspace Discriminant Subspace KNN	100 100 100 100 100 100	*Coronaviridae* *Coronaviridae* *Coronaviridae* *Coronaviridae* *Coronaviridae* *Coronaviridae*
Test-3(a\b)	29 COVID-19 virus sequences	Linear Discriminant Linear SVM Quadratic SVM Fine KNN Subspace Discriminant Subspace KNN	100 100 100 100 100 100	*Betacoronavirus* *Betacoronavirus* *Betacoronavirus* *Betacoronavirus* *Betacoronavirus* *Betacoronavirus*

Test-1 classified the COVID-19 virus as belonging to the realm Riboviria. The second test (Test-2) is designed to classify the COVID-19 virus among the families of the Riboviria realm. We completed the dataset pre-processing using the same rules as in Test-1 and obtained a dataset of 2779 sequences placed into the 12 families (*Betaflexiviridae*, *Bromoviridae*, *Caliciviridae*, *Coronaviridae*, *Flaviviridae*, *Peribunyaviridae*, *Phenuiviridae*, *Picornaviridae*, *Potyviridae*, *Reoviridae*, *Rhabdoviridae*, and *Secoviridae*), see Table 1. MLDSP-GUI with CGR at *k* = 7 as the numerical representation was used for the classification of the dataset in Test-2. The maximum classification accuracy of 93.1% is obtained using the Quadratic SVM model. The respective MoDMap3D is shown in Fig 1(b). All six classification models trained on 2779 sequences were used to classify (predict the label of) the 29 COVID-19 virus sequences. All of our machine learning-based models predicted the label as *Coronaviridae* for all 29 sequences (Table 2) with 100% classification accuracy. Test-2 correctly predicted the family of the COVID-19 virus sequences as *Coronaviridae*. Test-3 performs the genus-level classification.

The third test (Test-3a) is designed to classify the COVID-19 virus sequences at the genus level. We considered 208 *Coronaviridae* sequences available under four genera (*Alphacoronavirus*, *Betacoronavirus*, *Deltacoronavirus*, *Gammacoronavirus*) (Table 1). MLDSP-GUI with CGR at *k* = 7 as the numerical representation was used for the classification of the dataset in Test-3a. The maximum classification accuracy of 98.1% is obtained using the Linear Discriminant model and the respective MoDMap3D is shown in Fig 1(c). All six classification models trained on 208 sequences were used to classify (predict the label of) the 29 COVID-19 virus sequences. All of our machine learning-based models predicted the label as *Betacoronavirus* for all 29 sequences (Table 2). To verify that the correct prediction is not an artifact of possible bias because of larger *Betacoronavirus* cluster, we did a secondary Test-3b with cluster size limited to the size of smallest cluster (after removing the *Gammacoronavirus* because it just had 9 sequences). The maximum classification accuracy of 100% is obtained using the Linear Discriminant model for Test-3b. All six classification models trained on 60 sequences were used to classify the 29 COVID-19 virus sequences. All of our machine learning-based models predicted the label as *Betacoronavirus* for all 29 sequences (Table 2). This secondary test showed that the possible bias is not significant enough to have any impact on the classification performance.

**Fig 2:**
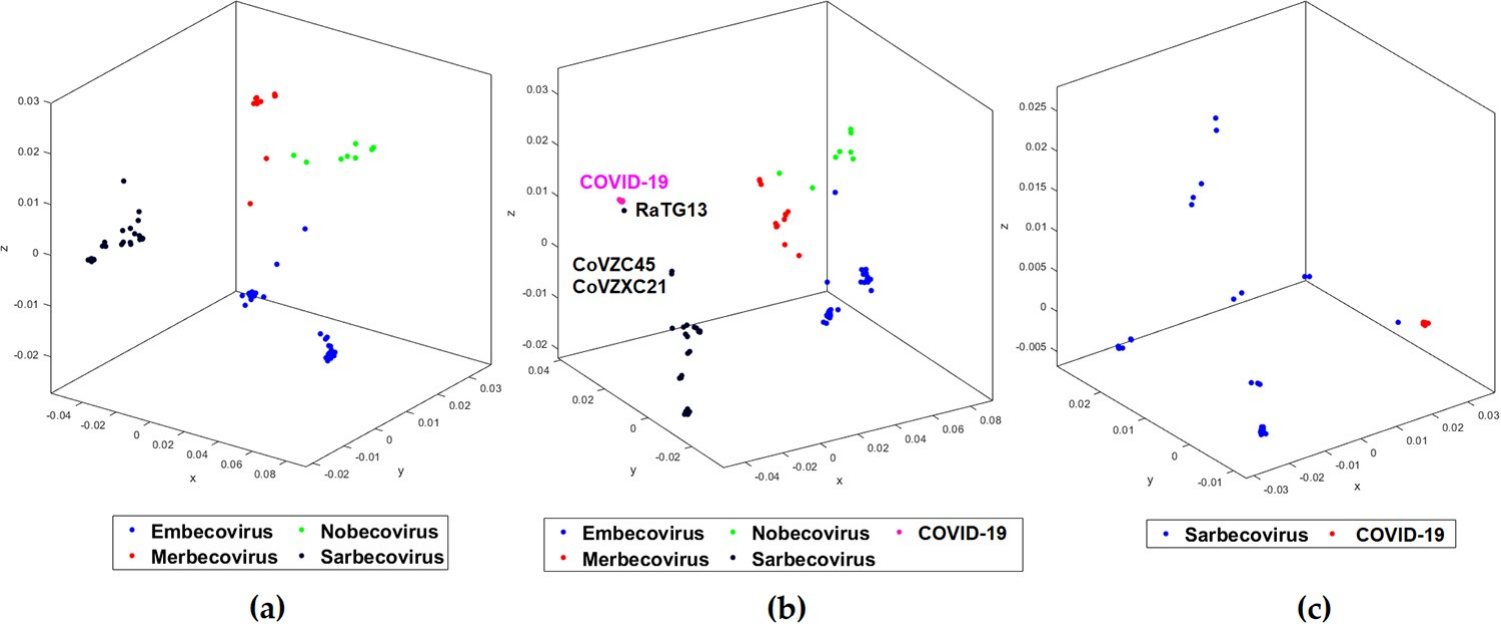
MoDMap3D of (a) 124 *Betacoronavirus* sequences from Test-4 classified into sub-genera, (b) 153 viral sequences from Test-5 classified into 4 sub-genera and COVID-19 virus, (c) 76 viral sequences from Test 6 classified into *Sarbecovirus* and COVID-19 virus.

Given confirmation that the COVID-19 virus belongs to the *Betacoronavirus* genus, there now is a question of its origin and relation to the other viruses of the same genus. To examine this question, we preprocessed our dataset from our third test to keep the sub-clusters of the *Betacoronavirus* with at least 10 sequences (Test-4). This gives 124 sequences placed into four clusters (*Embecovirus*, *Merbecovirus*, *Nobecovirus*, *Sarbecovirus*) (Table 3). The maximum classification accuracy of 98.4% with CGR at *k* = 7 as the numerical representation is obtained using the Quadratic SVM model. The respective MoDMap3D is shown in Fig 2(a). All six classifiers trained on 124 sequences predicted the label as *Sarbecovirus*, when used to predict the labels of 29 COVID-19 virus sequences. For Test-5, we added the COVID-19 virus with 29 sequences as the fifth cluster, see Table 3. The maximum classification accuracy of 98.7% with CGR at *k* = 7 as the numerical representation is obtained using the Subspace Discriminant model. The respective MoDMap3D is shown in Fig 2(b). In the MoDMap3D plot from Test-5, COVID-19 virus sequences are placed in a single distinct cluster, see Fig 2(b). As visually suggested by the MoDMap3D (Fig 2(b)), the average inter-cluster distances confirm that the COVID-19 virus sequences are closest to the *Sarbecovirus* (average distance 0.0556), followed by *Merbecovirus* (0.0746), *Embecovirus* (0.0914), and *Nobecovirus* (0.0916). The three closest sequences based on the average distances from all COVID-19 virus sequences are *RaTG13* (0.0203), *bat-SL-CoVZC45* (0.0418), and *bat-SL-CoVZXC21* (0.0428).

**Table 3:** Genus to sub-genus classification accuracy scores of *Betacoronavirus*.

Dataset	Clusters	Number of sequences	Classification model	Classification accuracy (in %)
Test-4: *Betacoronavirus*; 124 sequences; Maximum length: 31526 Minimum length: 29107 Median length: 30155 Mean length: 30300	Embecovirus Merbecovirus Nobecovirus Sarbecovirus	49 18 10 47	LinearDiscriminant LinearSVM QuadraticSVM FineKNN SubspaceDiscriminant SubspaceKNN AverageAccuracy	97.6 98.4 98.4 97.6 98.4 97.2 97.6
Test-5: *Betacoronavirus* and COVID-19 virus; 153 sequences; Maximum length: 31526 Minimum length: 29107 Median length: 29891 Mean length: 30217	*Embecovirus* *Merbecovirus* *Nobecovirus* *Sarbecovirus* COVID-19 virus	49 18 10 47 29	LinearDiscriminant LinearSVM QuadraticSVM FineKNN SubspaceDiscriminant SubspaceKNN AverageAccuracy	98.6 97.4 97.4 97.4 98.7 96.1 97.5
Test-6: *Sarbecovirus* and COVID-19 virus; 76 sequences; Maximum length: 30309 Minimum length: 29452 Median length: 29748 Mean length: 29772	*Sarbecovirus* COVID-19 virus	47 29	LinearDiscriminant LinearSVM QuadraticSVM FineKNN SubspaceDiscriminant SubspaceKNN AverageAccuracy	100 100 100 100 100 100 100

**Fig 3:**
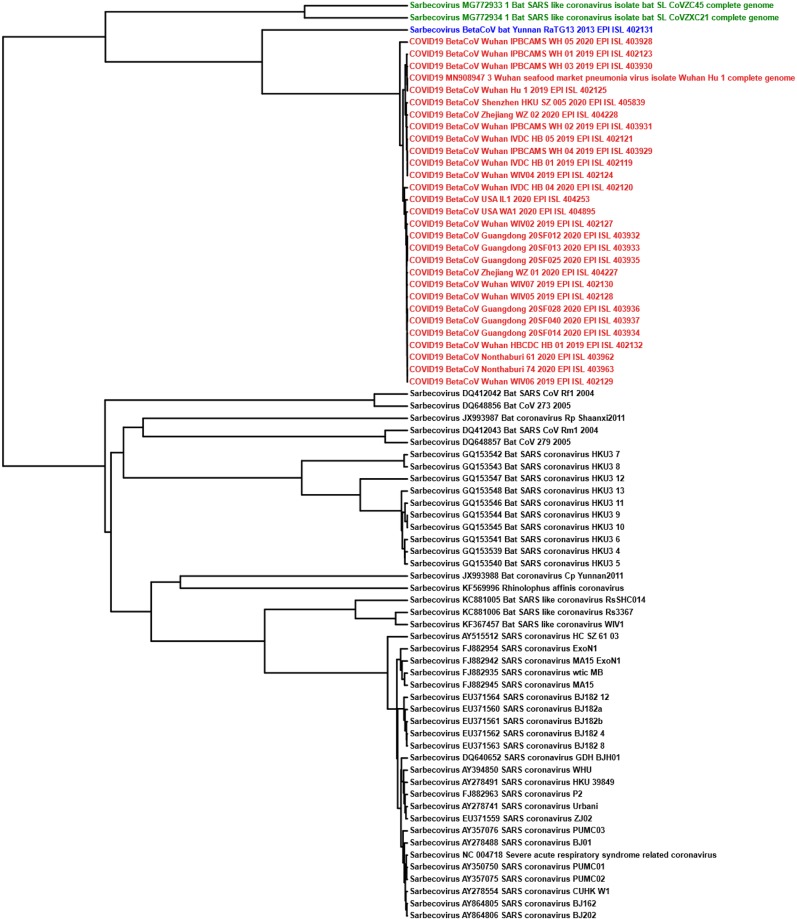
The UPGMA phylogenetic tree using the Pearson Correlation Coefficient generated pairwise distance matrix shows COVID-19 virus (Red) sequences proximal to the bat *Betacoronavirus RaTG13* (Blue) and bat SARS-like coronaviruses *ZC45/ZXC21* (Green) in a distinct lineage from the rest of *Sarbecovirus* sequences (Black).

**Fig 4:**
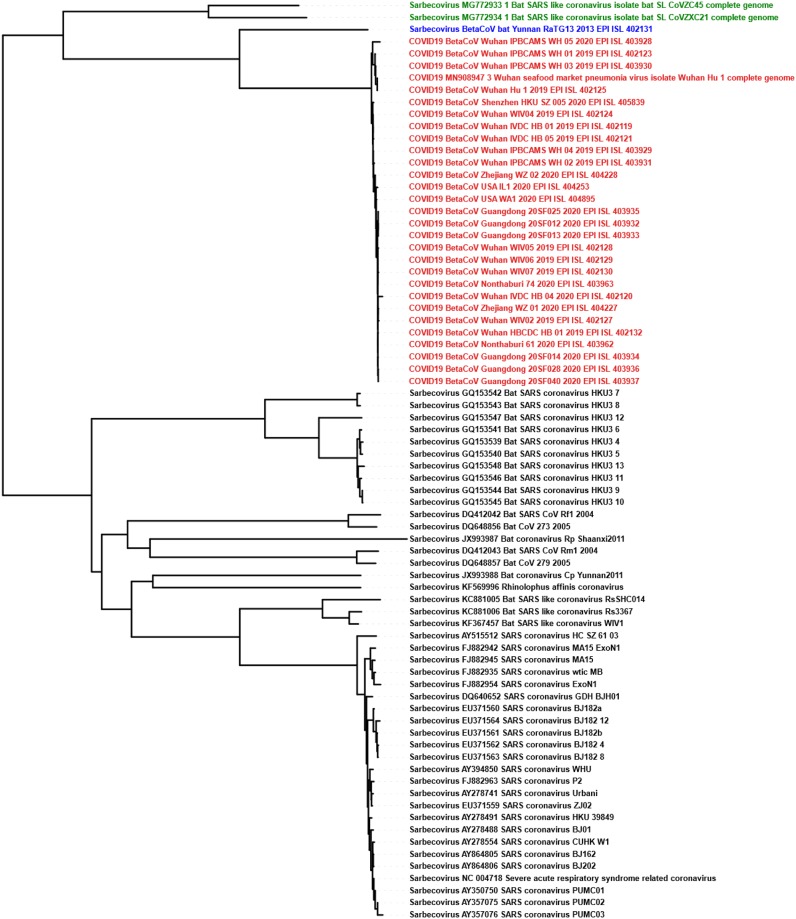
The neighbor-joining phylogenetic tree using the Pearson Correlation Coefficient generated pairwise distance matrix shows COVID-19 virus (Red) sequences proximal to the bat *Betacoronavirus RaTG13* (Blue) and bat SARS-like coronaviruses *ZC45/ZXC21* (Green) in a distinct lineage from the rest of *Sarbecovirus* sequences (Black).

For Test-6, we classified *Sarbecovirus* (47 sequences) and COVID-19 virus (29 sequences) clusters and achieved separation of the two clusters visually apparent in the MoDMap3D, see Fig 2(c). Quantitatively, using 10-fold cross-validation, all six of our classifiers report 100% classification accuracy. We generated phylogenetic trees (UPGMA and neighbor-joining) based on all pairwise distances for the dataset in Test-6 that show the separation of the two clusters and relationships within the clusters (Figs 3 and 4). As observed in Test-5, the phylogenetic trees show that the COVID-19 virus sequences are closer to the *Betacoronavirus* *RaTG13* sequence collected from a bat host.

**Fig 5:**
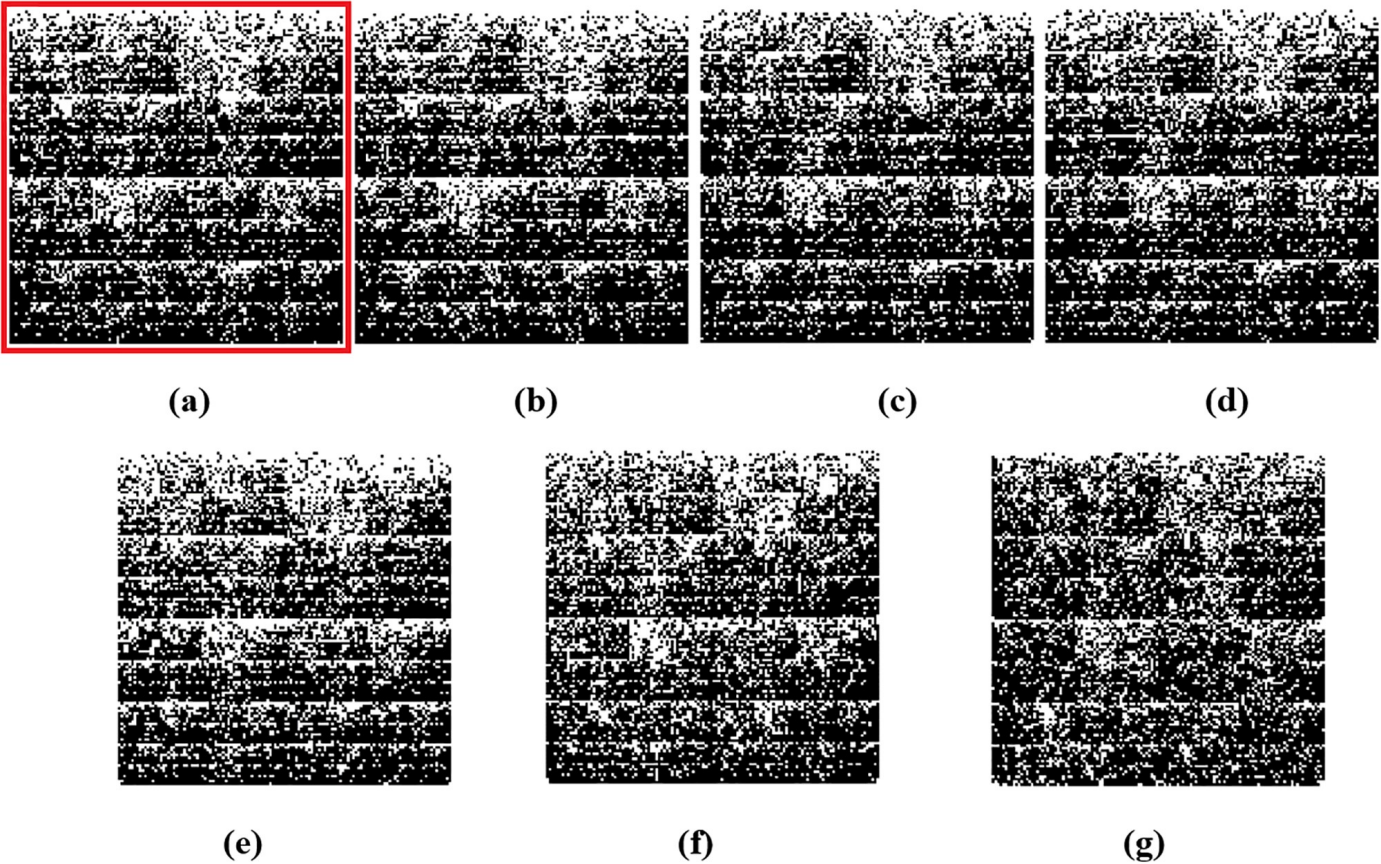
Chaos Game Representation (CGR) plots at *k* = 7 of (a) COVID-19 virus / Wuhan seafood market pneumonia virus isolate Wuhan-Hu-1/ *MN908947.3*, (b)*Betacoronavirus* / CoV / Bat / Yunnan /*RaTG13 / EPI_ISL_402131*, (c)*Betacoronavirus* / Bat SARS-like coronavirus isolate *bat-SL-CoVZC45 / MG772933.1*, (d)*Betacoronavirus* / Bat SARS-like coronavirus isolate *bat-SL-CoVZXC21 / MG772934.1*, (e)*Alphacoronavirus* /*DQ811787 PRCV ISU−1*, (f)*Gammacoronavirus* / Infectious bronchitis virus NGA /*A116E7 /2006/ FN430415*, and (g)*Deltacoronavirus* / PDCoV / USA / Illinois121 /2014/*KJ481931*. Chaos plot vertices are assigned top left Cytosine, top right Guanine, bottom left Adenine and bottom right Thymine.

Fig 5 shows the Chaos Game Representation (CGR) plots of different sequences from the four different genera (*Alphacoronavirus*, *Betacoronavirus*, *Deltacoronavirus*, *Gammacoronavirus*) of the family *Coronaviridae*. The CGR plots visually suggest and the pairwise distances confirm that the genomic signature of the COVID-19 virus Wuhan-Hu-1 (Fig 5(a)) is closer to the genomic signature of the *BetaCov-RaTG13* (Fig 5(b); distance: 0.0204), followed by the genomic signatures of *bat-SL-CoVZC45* (Fig 5(c); distance: 0.0417), *bat-SL-CoVZXC21*(Fig 5(d); distance: 0.0428), *Alphacoronavirus* /*DQ*811787 *PRCV* *ISU*-1 (Fig 5(e); distance: 0.0672), *Gammacoronavirus* / Infectious bronchitis virus NGA /*A*116*E*7/2006/*FN*430415 (Fig 5(f); distance: 0.0791), and *Deltacoronavirus* / PDCoV / USA / Illinois121 /2014/*KJ*481931 (Fig 5(g); distance: 0.0851).

**Fig 6:**
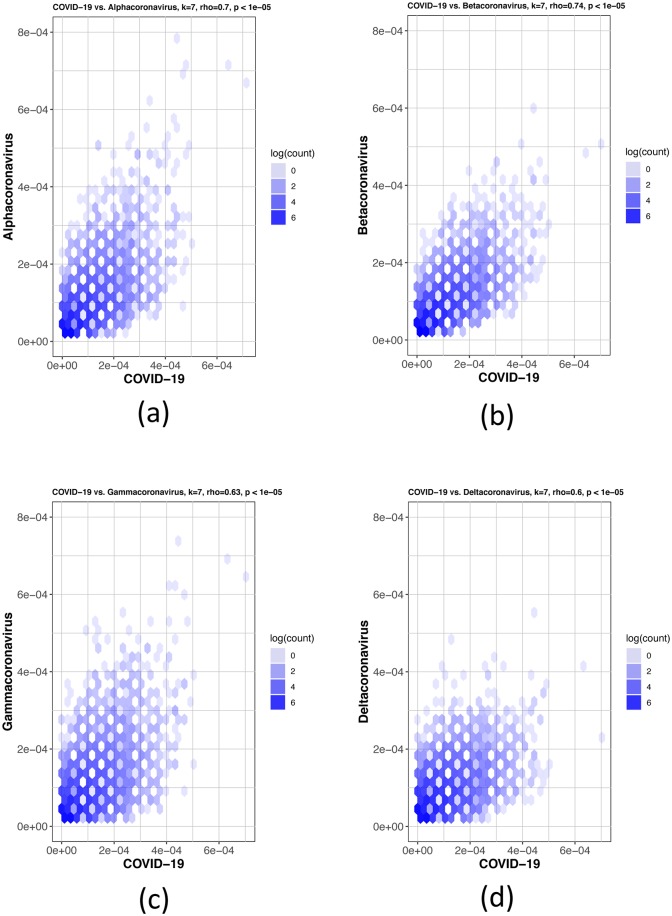
Hexbin scatterplots of the proportional *k*-mer (*k* = 7) frequencies of the COVID-19 virus sequences vs. the four genera: (a) *Alphacoronavirus*, *ρ* = 0.7; (b) *Betacoronavirus*, *ρ* = 0.74; (c) *Gammacoronavirus*, *ρ* = 0.63 and (d) *Deltacoronavirus*, *ρ* = 0.6. The color of each hexagonal bin in the plot represents the number of points (in natural logarithm scale) overlapping at that position. All *ρ* values resulted in *p*-values <10^−5^ for the correlation test. By visually inspecting each hexbin scatterplot, the degree of correlation is displayed by the variation in spread between the points. Hexagonal points that are closer together and less dispersed as seen in (b) are more strongly correlated and have less deviation.

**Fig 7:**
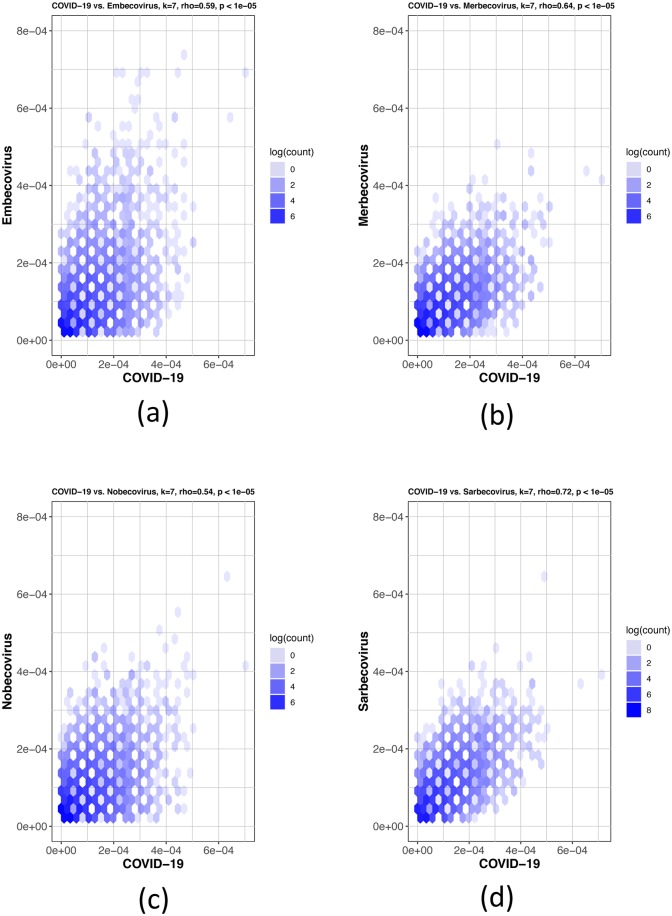
Hexbin scatterplots of the proportional *k*-mer (*k* = 7) frequencies of the COVID-19 virus sequences vs. the four sub-genera: (a) *Embecovirus*, *ρ* = 0.59; (b) *Merbecovirus*, *ρ* = 0.64; (c) *Nobecovirus*, *ρ* = 0.54 and (d) *Sarbecovirus*, *ρ* = 0.72. The color of each hexagonal bin in the plot represents the number of points (in natural logarithm scale) overlapping at that position. All *ρ* values resulted in *p*-values <10^−5^ for the correlation test. By visually inspecting each hexbin scatterplot, the degree of correlation is displayed by the variation in spread between the points. Hexagonal points that are closer together and less dispersed as seen in (d) are more strongly correlated and have less deviation.

The Spearman’s rank correlation coefficient tests were used to further confirm the MLDSP findings. The first test in Fig 6 shows COVID-19 virus being compared to the four genera; *Alphacoronavirus*, *Betacoronavirus*, *Gammacoronavirus* and *Deltacoronavirus*. The COVID-19 virus showed the highest *k*-mer frequency correlation to *Betacoronavirus* at *k* = 7 (Table 4), which is consistent with the MLDSP results in Test-3 (Table 2). The COVID-19 virus was then compared to all sub-genera within the *Betacoronavirus* genus: *Embecovirus*, *Merbecovirus*, *Nobecovirs* and *Sarbecovirus* seen in Fig 7. The Spearman’s rank test was again consistent with the MLDSP results seen in Table 3, as the *k*-mer frequencies at *k* = 7 showed the highest correlation to the sub-genus *Sarbecovirus* (Table 4). These tests confirm the findings in MLDSP and are consistent with the COVID-19 virus as part of the sub-genus *Sarbecovirus*.

**Table 4:** Spearman’s rank correlation coefficient (*ρ*) values from Figs 6 and 7, for which all *p*-values <10^−5^. The strongest correlation value was found between *Betacoronavirus* and *Sarbecovirus* when using the data sets from Test 3a from Table 2 and Test 4 from Table 3, respectively.

Dataset	Comparison Groups COVID-19 virus vs.	*ρ* value
Test-3a	*Alphacoronavirus*	0.70
	***Betacoronavirus***	**0.74**
	*Gammacoronavirus*	0.63
	*Deltacoronavirus*	0.60
Test-4	*Embecovirus*	0.59
	*Merbecovirus*	0.64
	*Nobecovirus*	0.54
	***Sarbecovirus***	**0.72**

## Discussion

Prior work elucidating the evolutionary history of the COVID-19 virus had suggested an origin from bats prior to zoonotic transmission [12, 33, 35, 38, 41, 62]. Most early cases of individuals infected with the COVID-19 virus had contact with the Huanan South China Seafood Market [26–31]. Human-to-human transmission is confirmed, further highlighting the need for continued intervention [33, 62–64]. Still, the early COVID-19 virus genomes that have been sequenced and uploaded are over 99% similar, suggesting these infections result from a recent cross-species event [12, 31, 40].

These prior analyses relied upon alignment-based methods to identify relationships between the COVID-19 virus and other coronaviruses with nucleotide and amino acid sequence similarities. When analyzing the conserved replicase domains of ORF1ab for coronavirus species classification, nearly 94% of amino acid residues were identical to SARS-CoV, yet overall genome similarity was only around 70%, confirming that the COVID-19 virus was genetically different [64]. Within the RdRp region, it was found that another bat coronavirus, *RaTG13*, was the closest relative to the COVID-19 virus and formed a distinct lineage from other bat SARS-like coronaviruses [38, 40]. Other groups found that two bat SARS-like coronaviruses, *bat-SL-CoVZC45* and *bat-SL-CoVZXC21*, were also closely related to the COVID-19 virus [12, 33–37]. There is a consensus that these three bat viruses are most similar to the COVID-19 virus, however, whether or not the COVID-19 virus arose from a recombination event is still unknown [38–40].

Regardless of the stance on recombination, current consensus holds that the hypothesis of the COVID-19 virus originating from bats is highly likely. Bats have been identified as a reservoir of mammalian viruses and cross-species transmission to other mammals, including humans [4, 7, 8, 10, 13, 65–67]. Prior to intermediary cross-species infection, the coronaviruses SARS-CoV and MERS-CoV were also thought to have originated in bats [24, 25, 34, 68–70]. Many novel SARS-like coronaviruses have been discovered in bats across China, and even in European, African and other Asian countries [34, 71–77]. With widespread geographic coverage, SARS-like coronaviruses have likely been present in bats for a long period of time and novel strains of these coronaviruses can arise through recombination [4]. Whether or not the COVID-19 virus was transmitted directly from bats, or from intermediary hosts, is still unknown, and will require identification of the COVID-19 virus in species other than humans, notably from the wet market and surrounding area it is thought to have originated from [30]. While bats have been reported to have been sold at the Huanan market, at this time, it is still unknown if there were intermediary hosts involved prior to transmission to humans [27, 31, 33, 39, 78]. Snakes had been proposed as an intermediary host for the COVID-19 virus based on relative synonymous codon usage bias studies between viruses and their hosts [39], however, this claim has been disputed [79]. China CDC released information about environmental sampling in the market and indicated that 33 of 585 samples had evidence of the COVID-19 virus, with 31 of these positive samples taken from the location where wildlife booths were concentrated, suggesting possible wildlife origin [80, 81]. Detection of SARS-CoV in Himalyan palm civets and horseshoe bats identified 29 nucleotide sequences that helped trace the origins of SARS-CoV isolates in humans to these intermediary species [13, 24, 38, 77]. Sampling additional animals at the market and wildlife in the surrounding area may help elucidate whether intermediary species were involved or not, as was possible with the SARS-CoV.

Viral outbreaks like COVID-19 demand timely analysis of genomic sequences to guide the research in the right direction. This problem being time-sensitive requires quick sequence similarity comparison against thousands of known sequences to narrow down the candidates of possible origin. Alignment-based methods are known to be time-consuming and can be challenging in cases where homologous sequence continuity cannot be ensured. It is challenging (and sometimes impossible) for alignment-based methods to compare a large number of sequences that are too different in their composition. Alignment-free methods have been used successfully in the past to address the limitations of the alignment-based methods [48–51]. The alignment-free approach is quick and can handle a large number of sequences. Moreover, even the sequences coming from different regions with different compositions can be easily compared quantitatively, with equally meaningful results as when comparing homologous/similar sequences. We use MLDSP-GUI (a variant of MLDSP with additional features), a machine learning-based alignment-free method successfully used in the past for sequence comparisons and analyses [50]. The main advantage alignment-free methodology offers is the ability to analyze large datasets rapidly. In this study we confirm the taxonomy of the COVID-19 virus and, more generally, propose a method to efficiently analyze and classify a novel unclassified DNA sequence against the background of a large dataset. We namely use a “decision tree” approach (paralleling taxonomic ranks), and start with the highest taxonomic level, train the classification models on the available complete genomes, test the novel unknown sequences to predict the label among the labels of the training dataset, move to the next taxonomic level, and repeat the whole process down to the lowest taxonomic label.

Test-1 starts at the highest available level and classifies the viral sequences to the 11 families and Riboviria realm (Table 1). There is only one realm available in the viral taxonomy, so all of the families that belong to the realm Riboviria are placed into a single cluster and a random collection of 500 sequences are selected. No realm is defined for the remaining 11 families. The objective is to train the classification models with the known viral genomes and then predict the labels of the COVID-19 virus sequences. The maximum classification accuracy score of 95% was obtained using the Quadratic SVM model. This test demonstrates that MLDSP-GUI can distinguish between different viral families. The trained models are then used to predict the labels of 29 COVID-19 virus sequences. As expected, all classification models correctly predict that the COVID-19 virus sequences belong to the Riboviria realm, see Table 2. Test-2 is composed of 12 families from the Riboviria, see Table 1, and the goal is to test if MLDSP-GUI is sensitive enough to classify the sequences at the next lower taxonomic level. It should be noted that as we move down the taxonomic levels, sequences become much more similar to one another and the classification problem becomes challenging. MLDSP-GUI is still able to distinguish between the sequences within the Riboviria realm with a maximum classification accuracy of 91.1% obtained using the Linear Discriminant classification model. When the COVID-19 virus sequences are tested using the models trained on Test-2, all of the models correctly predict the COVID-19 virus sequences as *Coronaviridae* (Table 2). Test-3a moves down another taxonomic level and classifies the *Coronaviridae* family to four genera (*Alphacoronavirus*, *Betacoronavirus*, *Deltacoronavirus*, *Gammacoronavirus*), see Table 1. MLDSP-GUI distinguishes sequences at the genus level with a maximum classification accuracy score of 98%, obtained using the Linear Discriminant model. This is a very high accuracy rate considering that no alignment is involved and the sequences are very similar. All trained classification models correctly predict the COVID-19 virus as *Betacoronavirus*, see Table 2. Test-3a has *Betacoronavirus* as the largest cluster and it can be argued that the higher accuracy could be a result of this bias. To avoid bias, we did an additional test removing the smallest cluster *Gammacoronavirus* and limiting the size of remaining three clusters to the size of the cluster with the minimum number of sequences i.e. 20 with Test-3b. MLDSP-GUI obtains 100% classification accuracy for this additional test and still predicts all of the COVID-19 virus sequences as *Betacoronavirus*. These tests confirm that the COVID-19 virus sequences are from the genus *Betacoronavirus*.

Sequences become very similar at lower taxonomic levels (sub-genera and species). Test-4, Test-5, and Test-6 investigate within the genus *Betacoronavirus* for sub-genus classification. Test-4 is designed to classify *Betacoronavirus* into the four sub-genera (*Embecovirus*, *Merbecovirus*, *Nobecovirus*, *Sarbecovirus*), see Table 3. MLDSP-GUI distinguishes sequences at the sub-genus level with a maximum classification accuracy score of 98.4%, obtained using the Quadratic SVM model. All of the classification models trained on the dataset in Test-4 predicted the label of all 29 COVID-19 virus sequences as *Sarbecovirus*. This suggests substantial similarity between the COVID-19 virus and the *Sarbecovirus* sequences. Test-5 and Test-6 (see Table 3) are designed to verify that the COVID-19 virus sequences can be differentiated from the known species in the *Betacoronavirus* genus. MLDSP-GUI achieved a maximum classification score of 98.7% for Test-5 and 100% for Test-6 using Subspace Discriminant classification model. This shows that although the COVID-19 virus and *Sarbecovirus* are closer on the basis of genomic similarity (Test-4), they are still distinguishable from known species. Therefore, these results suggest that the COVID-19 virus may represent a genetically distinct species of *Sarbecovirus*. All the COVID-19 virus sequences are visually seen in MoDMap3D generated from Test-5 (see Fig 2(b)) as a closely packed cluster and it supports a fact that there is 99% similarity among these sequences [12, 31]. The MoDMap3D generated from the Test-5 (Fig 2(b)) visually suggests and the average distances from COVID-19 virus sequences to all other sequences confirm that the COVID-19 virus sequences are most proximal to the *RaTG13* (distance: 0.0203), followed by the *bat-SL-CoVZC45* (0.0418), and *bat-SL-CoVZX21* (0.0428). To confirm this proximity, UPGMA and neighbor-joining phylogenetic trees are computed from the PCC-based pairwise distance matrix of sequences in Test-6, see Figs 3 and 4. Notably, the UPGMA model assumes that all lineages are evolving at a constant rate (equal evolution rate among branches). This method may produce unreliable results in cases where the genomes of some lineages evolve more rapidly than those of the others. To further verify the phylogenetic relationships, we also produced a phylogenetic tree using the neighbor-joining method that allows different evolution rates among branches and obtained a highly similar output. The phylogenetic trees placed the *RaTG13* sequence closest to the COVID-19 virus sequences, followed by the *bat-SL-CoVZC45* and *bat-SL-CoVZX21* sequences. This closer proximity represents the smaller genetic distances between these sequences and aligns with the visual sequence relationships shown in the MoDMap3D of Fig 2(b).

We further confirm our results regarding the closeness of the COVID-19 virus with the sequences from the *Betacoronavirus* genus (especially sub-genus *Sarbecovirus*) by a quantitative analysis based on the Spearman’s rank correlation coefficient tests. Spearman’s rank correlation coefficient [58–61] tests were applied to the frequencies of oligonucleotide segments, adjusting for the total number of segments, to measure the degree and statistical significance of correlation between two sets of genomic sequences. Spearman’s *ρ* value provides the degree of correlation between the two groups and their *k*-mer frequencies. The COVID-19 virus was compared to all genera under the *Coronaviridae* family and the *k*-mer frequencies showed the strongest correlation to the genus *Betacoronavirus*, and more specifically *Sarbecovirus*. The Spearman’s rank tests corroborate that the COVID-19 virus is part of the *Sarbecovirus* sub-genus, as shown by CGR and MLDSP. When analyzing sub-genera, it could be hard to classify at lower *k* values due to the short oligonucleotide frequencies not capturing enough information to highlight the distinctions. Therefore despite the Spearman’s rank correlation coefficient providing results for *k* = 1 to *k* = 7, the higher *k*-mer lengths provided more accurate results, and *k* = 7 was used.

Attributes of the COVID-19 virus genomic signature are consistent with previously reported mechanisms of innate immunity operating in bats as a host reservoir for coronaviruses. Vertebrate genomes are known to have an under-representation of CG dinucleotides in their genomes, otherwise known as CG suppression [82, 83]. This feature is thought to have been due to the accumulation of spontaneous deamination mutations of methyl-cytosines over time [82]. As viruses are obligate parasites, evolution of viral genomes is intimately tied to the biology of their hosts [84]. As host cells develop strategies such as RNA interference and restriction-modification systems to prevent and limit viral infections, viruses will continue to counteract these strategies [83–85]. Dinucleotide composition and biases are pervasive across the genome and make up a part of the organism’s genomic signature [84]. These host genomes have evolutionary pressures that shape the host genomic signature, such as the pressure to eliminate CG dinucleotides within protein coding genes in humans [83]. Viral genomes have been shown to mimic the same patterns of the hosts, including single-stranded positive-sense RNA viruses, which suggests that many RNA viruses can evolve to mimic the same features of their host’s genes and genomic signature [82–86]. As genomic composition, specifically in mRNA, can be used as a way of discriminating self vs non-self RNA, the viral genomes are likely shaped by the same pressures that influence the host genome [83]. One such pressure on DNA and RNA is the APOBEC family of enzymes, members of which are known to cause G to A mutations [86–88]. While these enzymes primarily work on DNA, it has been demonstrated that these enzymes can also target RNA viral genomes [87]. The APOBEC enzymes therefore have RNA editing capability and may help contribute to the innate defence system against various RNA viruses [86]. This could therefore have a direct impact on the genomic signature of RNA viruses. Additional mammalian mechanisms for inhibiting viral RNA have been highlighted for retroviruses with the actions of zinc-finger antiviral protein (ZAP) [82]. ZAP targets CG dinucleotide sequences, and in vertebrate host cells with the CG suppression in host genomes, this can serve as a mechanism for the distinction of self vs non-self RNA and inhibitory consequences [82]. Coronaviruses have A/U rich and C/G poor genomes, which over time may have been, in part, a product of cytidine deamination and selection against CG dinucleotides [89–91]. This is consistent with the fact that bats serve as a reservoir for many coronaviruses and that bats have been observed to have some of the largest and most diverse arrays of APOBEC genes in mammals [67, 69]. The Spearman’s rank correlation data and the patterns observed in the CGR images from Fig 5, of the coronavirus genomes, including the COVID-19 virus identify patterns such as CG underepresentation, also present in vertebrate and, importantly, bat host genomes.

With human-to-human transmission confirmed and concerns for asymptomatic transmission, there is a strong need for continued intervention to prevent the spread of the virus [32, 33, 62–64]. Due to the high amino acid similarities between the COVID-19 virus and SARS-CoV main protease essential for viral replication and processing, anticoronaviral drugs targeting this protein and other potential drugs have been identified using virtual docking to the protease for treatment of COVID-19 [29, 43, 44, 92–95]. The human ACE2 receptor has also been identified as the potential receptor for the COVID-19 virus and represents a potential target for treatment [41, 42].

**Fig 8:**
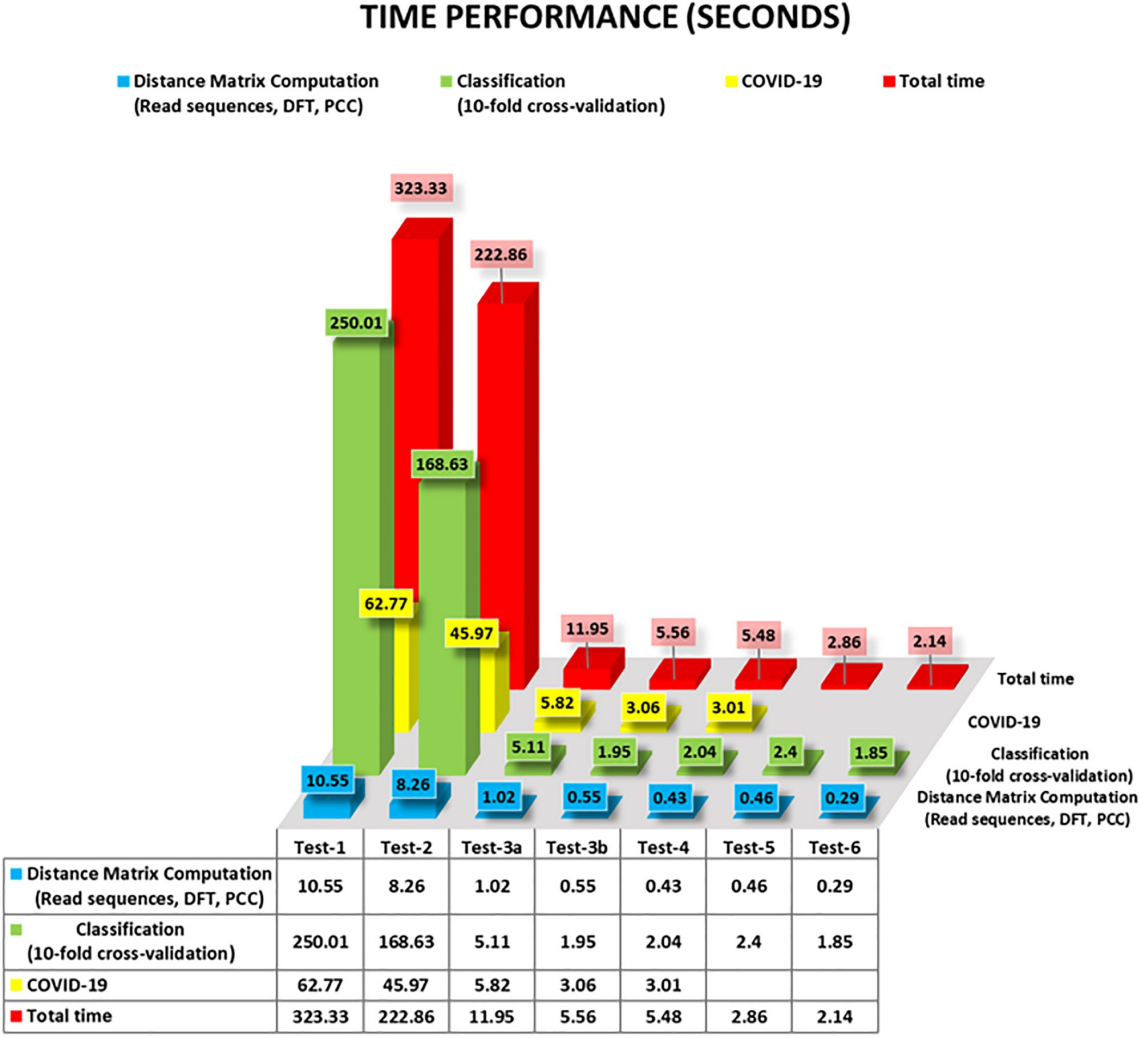
Time performance of MLDSP-GUI for Test1 to Test-6 (in seconds).

MLDSP-GUI is an ultra-fast, alignment-free method as is evidenced by the time-performance of MLDSP-GUI for Test-1 to Test-6 given in Fig 8. MLDSP-GUI took just 10.55 seconds to compute a pairwise distance matrix (including reading sequences, computing magnitude spectra using DFT, and calculating the distance matrix using PCC combined) for the Test-1 (largest dataset used in this study with 3273 complete genomes). All of the tests combined (Test-1 to Test-6) are doable in under 10 minutes including the computationally heavy 10-fold cross-validation, and testing of the 29 COVID-19 virus sequences.

The results of our machine learning-based alignment-free analyses using MLDSP-GUI support the hypothesis of a bat origin for the COVID-19 virus and classify COVID-19 virus as sub-genus *Sarbecovirus*, within *Betacoronavirus*.

## Conclusion

This study provides an alignment-free method based on intrinsic genomic signatures that can deliver highly-accurate real-time taxonomic predictions of yet unclassified new sequences, *ab initio*, using raw DNA sequence data alone and without the need for gene or genome annotation. We use this method to provide evidence for the taxonomic classification of the COVID-19 virus as *Sarbecovirus*, within *Betacoronavirus*, as well as quantitative evidence supporting a bat origin hypothesis. Our results are obtained through a comprehensive analysis of over 5000 unique viral sequences, through an alignment-free analysis of their two-dimensional genomic signatures, combined with a “decision tree” use of supervised machine learning and confirmed by Spearman’s rank correlation coefficient analyses. This study suggests that such alignment-free approaches to comparative genomics can be used to complement alignment-based approaches when timely taxonomic classification is of the essence, such as at critical periods during novel viral outbreaks.

## Supporting information

S1 File (PDF)
