# Correction: Machine learning using intrinsic genomic signatures for rapid classification of novel pathogens: COVID-19 case study

**DOI:** 10.1371/journal.pone.0246465

**Published:** 2021-01-27

**Authors:** The PLOS ONE Staff

## Notice of republication

This article was republished on May 15, 2020, to correct errors in the figure captions that were introduced during the typesetting process. The publisher apologizes for the errors. Please download this article again to view the correct version.
